# A high-resolution Orbitrap Mass spectral library for trace volatile compounds in fruit wines

**DOI:** 10.1038/s41597-022-01594-x

**Published:** 2022-08-13

**Authors:** Yaran Liu, Na Li, Xiaoyao Li, Wenchao Qian, Jiani Liu, Qingyu Su, Yixin Chen, Bolin Zhang, Baoqing Zhu, Jinxin Cheng

**Affiliations:** 1 Beijing Key Laboratory of Forestry Food Processing and Safety, Department of Food Science, College of Biological Sciences and Biotechnology, Beijing Forestry University, Beijing, 100083 China; 2 School of Cyberspace Security, Beijing University of Posts and Telecommunications, Beijing, 100876 China; 3 China People’s Police University, Hebei, 065000 China

**Keywords:** Agriculture, Inorganic chemistry

## Abstract

The overall aroma is an important factor of the sensory quality of fruit wines, which attributed to hundreds of volatile compounds. However, the qualitative determination of trace volatile compounds is considered to be very challenging work. GC-Orbitrap-MS with high resolution and high sensitivity provided more possibilities for the determination of volatile compounds, but without the high-resolution mass spectral library. For accuracy of qualitative determination in fruit wines by GC-Orbitrap-MS, a high-resolution mass spectral library, including 76 volatile compounds, was developed in this study. Not only the HRMS spectrum but also the exact ion fragment, relative abundance, retention indices (RI), CAS number, chemical structure diagram, aroma description and aroma threshold (ortho-nasally) were provided and were shown in a database website (Food Flavor Laboratory, http://foodflavorlab.cn/). HRMS library was used to successfully identify the volatile compounds mentioned above in 16 fruit wines (5 blueberry wines, 6 goji berry wines and 5 hawthorn wines). The library was developed as an important basis for further understanding of trace volatile compounds in fruit wines.

## Abstract (summary)

**Table:** 

Measurement(s)	volatile compounds
Technology Type(s)	GC-Orbitrap-MS

## Background & Summary

Among the hundreds of volatile compounds detected in fruit wines, only a small percentage of them could play key roles in the contribution of characteristic aroma^1^. Currently, the gas chromatograph-mass spectrometer has been widely used for the identification and quantification of aroma compounds. The quadrupole mass spectrometer (qMS) could be the most common mass spectrometer for analysis^2–6^. However, some trace analytes were difficult to be detected using qMS due to their low resolution and sensitivity^4,7–12^. These trace compounds needed to be identified by other detectors. The aldehydes and ketones could be detected in Syrah wines^13^ and model wine solution by flame ionization detector (FID)^14,15^. The flame photometry (FPD) was used to identify sulfur compounds in Cabernet Sauvignon wines^16,17^. Besides, sulphur chemiluminescence (SCD)^13,18,19^ and pulsed flame photometry (PFPD)^20,21^ also could be used for the analysis of sulfur compounds in grape wines. The pyrazines could be identified in wines^22^ and oak woods^23^ by nitrogen-phosphorous detection (NPD). The triple-quadrupole mass spectrometer (QqQ-MS) in selected-reaction-monitoring (SRM) could identify lactones^24^, terpenes^25^ and sulfur compounds^26^ in wines. Thus, multiple methods had to be used for the detection of various aroma compounds^14,16^. Meanwhile, the use of multiple instruments is time-consuming and costly. And it is also difficult to have so many instruments in a same laboratory. And it is an urgent challenge to identify trace aroma volatile compounds mentioned above simply and effectively in fruit wines.

In recent years, high-resolution mass spectrometry, such as quadrupole-time-of-flight-MS (Q-TOF), could improve the accuracy of identification^22,23,27^. Since Orbitrap-MS technology invented by Alexander Makarov was first commercially available in 2005, this new technique of high resolution and high sensitivity mass spectrometry has been shown great advantages for qualitative and quantitative analysis of compounds^28–30^, and therefore many studies have been focused on metabolomics using liquid chromatography coupling^31–34^. After GC was coupled with Orbitrap-MS in 2015, its resolution could reach 60,000 (219 m/z, FWHM), mass accuracy could reach 1 ppm, and sensitivity could reach femtogram level, which provided more possibilities to advance the depth and breadth of GC-MS technology^35,36^. At present, GC-Orbitrap-MS began to be used to detect pesticide residues^37^, nitrosamines in children’s products^38^, persistent organic pollutants in the environment^39^, soluble and extractable substances in package materials^40^, stimulants and banned substances in urine^41^ and metabonomics^42^. GC-Orbitrap-MS can provide accurate qualitative quantification of benzene compounds in chili peppers^43^. In summary, the GC-Orbitrap-MS could be a potential technique for the determination of aroma volatile compounds in fruit wines due to its high resolution and high sensitivity.

At present, the NIST library is widely used for the identification of aroma volatile compounds analyzed by gas chromatography-mass spectrometry^7,8,44,45^. However, the mass spectrums in the NIST library were mostly obtained by low-resolution mass spectrometry. There were differences in ion fragments and ion abundance between high-resolution mass spectrums obtained by GC-Orbitrap-MS and low-resolution mass spectrums obtained by GC-Quadrupole-MS^46^, which led to the qualitative inaccuracy. The high-resolution mass spectrometry (HRMS) spectrums of aroma compounds analyzed by GC-Orbitrap-MS need to be established for accurate identification. In addition, the basic information of aroma compounds, such as CAS number, chemical structure diagram, aroma description and aroma threshold (ortho-nasally), need to be acquired by a large collection of literature. Thus, there is an urgent need to establish a library of HRMS spectrum and basic information to facilitate analyzing and consulting by scholars all over the world.

## Methods

### Overview of the experimental design

#### Materials and methods

##### Chemical and reagents

The information of standards was shown in Table 1. The individual stock solution of each standard is dissolved in ethanol and stored at −20 °C.

**Table 1:** The information of standards used in this study.

Compounds	CAS No.	Purity	Manufacturer	Formula	RI	Content^h^/μg.L^−1^
**Ester**						
Ethyl butanoate	105-54-4	≥99.5%	Aladdin^b^	C_6_H_12_O_2_	1065	10020
Ethyl 2-methylbutanoate	7452-79-1	>98.0%	Aladdin	C_7_H_14_O_2_	1077	5030
Ethyl isovalerate	108-64-5	>99.0%	Adamas^c^	C_7_H_14_O_2_	1093	11050
Isoamyl acetate	123-92-2	≥99.5%	Macklin	C_7_H_14_O_2_	1139	11390
Methyl caproate	106-70-7	>99.0%	Macklin	C_7_H_14_O_2_	1200	5120
Ethyl hexanoate	123-66-0	>99.0%	Aladdin	C_8_H_16_O_2_	1243	30300
Ethyl heptanoate	106-30-9	≥99.5%	Macklin	C_9_H_18_O_2_	1340	5050
Ethyl lactate	97-64-3	≥99.0%	Macklin	C_5_H_10_O_3_	1350	50810
Heptyl acetate	112-06-1	≥98.0%	TCI	C_9_H_18_O_2_	1380	3280
Methyl octanoate	111-11-5	≥99.0%	Adamas	C_9_H_18_O_2_	1394	2000
Ethyl caprylate	106-32-1	>99.0%	Aladdin	C_10_H_20_O_2_	1439	29670
Ethyl 3-hydroxybutyrate	5405-41-4	>99.0%	Macklin	C_6_H_12_O_3_	1511	15170
Ethyl nonanoate	123-29-5	≥95.0%	Macklin	C_11_H_22_O_2_	1521	7250
Ethyl 2-hydroxy-4-methylpentanoate	10348-47-7	≥98.0%	Aladdin	C_8_H_16_O_3_	1525	10180
Ethyl caprate	110-38-3	>99.0%	Macklin	C_12_H_24_O_2_	1572	20980
Ethyl succinate	123-25-1	≥99.5%	Macklin	C_8_H_14_O_4_	1592	50360
Methyl salicylate	119-36-8	≥99.5%	Macklin	C_8_H_8_O_3_	1675	4760
Ethyl benzeneacetate	101-97-3	≥99.5%	Aladdin	C_10_H_12_O_2_	1689	1940
Ethyl salicylate	118-61-6	>99.0%	Aladdin	C_9_H_10_O_3_	1710	5480
Ethyl hydrocinnamate	2021-28-5	>98.0%	TCI^d^	C_11_H_14_O_2_	1785	12040
Ethyl cinnamate	103-36-6	>98.0%	Adamas	C_11_H_12_O_2_	2031	5040
Monoethyl succinate	1070-34-4	>95.0%	Aladdin	C_6_H_10_O_4_	2308	11020
**Carbonyl compounds**						
(*E*)-2-Hexenal	6728-26-3	>98.0%	Aladdin	C_6_H_10_O	1329	7120
(*E*)-2-Heptenal	18829-55-5	>95.0%	Aladdin	C_7_H_12_O	1362	6530
(*E*)-2-Octenal	2548-87-0	>95.0%	Macklin	C_8_H_14_O	1432	1900
(*E*,*E*)-2,4-Heptadienal	4313-03-5	>90.0%	Macklin	C_7_H_10_O	1498	10220
(*E*,*Z*)-2,6-Nonadienal	557-48-2	≥95.0%	Aladdin	C_9_H_14_O	1545	4160
Benzeneacetaldehyde	122-78-1	>95.0%	Macklin	C_8_H_8_O	1574	5720
**High alcohols**						
Isobutanol	78-83-1	≥99.5%	Aladdin	C_4_H_10_O	1112	20620
Isoamylol	123-51-3	≥99.5%	Aladdin	C_5_H_12_O	1217	78820
1-Pentanol	71-41-0	≥99.5%	Macklin	C_5_H_12_O	1259	6340
2-Heptanol	543-49-7	>98.0%	Aladdin	C_7_H_16_O	1327	8700
3-Octenol	3391-86-4	>98.0%	Aladdin	C_8_H_16_O	1456	3220
1-Heptanol	111-70-6	>95.0%	Macklin	C_7_H_16_O	1460	5490
2-Nonanol	628-99-9	≥98.0%	Aladdin	C_9_H_20_O	1511	3240
1-Octanol	111-87-5	≥99.5%	Macklin	C_8_H_18_O	1532	9060
2-Phenylethanol	60-12-8	≥99.5%	Aladdin	C_8_H_10_O	1817	50690
2-Phenoxyethanol	122-99-6	≥ 99.5%	Macklin	C_8_H_10_O_2_	2043	9900
**Lactone**						
γ-Octalactone	104-50-7	>98.0%	Sigma-Aldrich	C_11_H_20_O_2_	1814	4140
δ-Octalactone	698-76-0	>98.0%	Sigma-Aldrich	C_8_H_14_O_2_	1862	3480
γ-Nonalactone	104-61-0	>98.0%	Sigma-Aldrich	C_8_H_14_O_2_	1925	3260
Pantolactone	599-04-2	>99.0%	Sigma-Aldrich	C_6_H_10_O_3_	1935	19860
γ-Decalactone	706-14-9	>98.0%	Sigma-Aldrich	C_10_H_18_O_2_	2041	3700
Sotolon	28664-35-9	>97.0%	Sigma-Aldrich	C_6_H_8_O_3_	2108	4980
γ-Undecalactone	104-67-6	>98.0%	Sigma-Aldrich	C_11_H_20_O_2_	2161	3520
**Acid**						
Butanoic acid	107-92-6	≥99.5%	Sigma-Aldrich	C_4_H_8_O_2_	1574	30960
Hexanoic acid	142-62-1	≥99.5%	Macklin	C_6_H_12_O_2_	1762	25780
Ethylhexanoic acid	149-57-5	≥99.9%	Aladdin	C_8_H_16_O_2_	1860	10090
Octanoic acid	124-07-2	≥99.5%	Aladdin	C_8_H_16_O_2_	1973	56880
Decanoic acid	334-48-5	>99.0%	Aladdin	C_10_H_20_O_2_	2190	20170
Benzoic acid	65-85-0	≥99.9%	Aladdin	C_7_H_6_O_2_	2378	11630
**Pyrazine**						
3-Isopropyl-2-methoxypyrazine	25773-40-4	>97.0%	Sigma-Aldrich	C_8_H_12_ON_2_	1435	1280
2-sec-Butyl-3-Methoxypyrazine	24168-70-5	>99.0%	Sigma-Aldrich	C_9_H_14_ON_2_	1453	980
5-Ethyl-2,3-dimethylpyrazine	15707-34-3	>98.0%	Sigma-Aldrich	C_8_H_12_N_2_	1459	2230
2-Isobutyl-3-methoxypyrazine	24683-00-9	>99.0%	Sigma-Aldrich	C_9_H_14_ON_2_	1513	1490
Acetylpyrazine	22047-25-2	>97.0%	Sigma-Aldrich	C_6_H_6_N_2_O	1565	2010
**Furan**						
Furfural	98-01-1	>99.0%	Sigma-Aldrich	C_5_H_4_O_2_	1472	5250
Acetylfuran	1192-62-7	>99.0%	Sigma-Aldrich	C_6_H_6_O_2_	1505	9840
5-Methylfurfural	620-02-0	>99.0%	Sigma-Aldrich	C_6_H_6_O_2_	1540	1740
Ethyl 2-furoate	614-99-3	>99.0%	Sigma-Aldrich	C_7_H_8_O_3_	1565	4250
Furfuryl alcohol	98-00-0	>98.0%	Sigma-Aldrich	C_5_H_6_O_2_	1585	10820
5-Hydroxymethylfurfural	67-47-0	>99.0%	Sigma-Aldrich	C_6_H_6_O_3_	2415	20050
**Terpenes**						
D-Limonene	5989-27-5	≥99.0%	TCI	C_10_H_16_	1203	1860
Terpinolene	586-62-9	>90.0%	TCI	C_10_H_16_	1284	2330
β-Linalool	78-70-6	>98.0%	Macklin	C_10_H_18_O	1527	2410
Citronellyl acetate	150-84-5	≥95.0%	Aladdin	C_12_H_22_O_2_	1583	3180
β-Ionone	14901-07-6	>97.0%	Aladdin	C_13_H_20_O	1833	1560
**Benzene**						
*o*-Xylene	95-47-6	≥99.0%	Macklin	C_8_H_10_	1192	1520
Styrene	100-42-5	≥99.5%	Macklin	C_8_H_8_	1264	2190
*p*-Cymene	99-87-6	≥99.5%	Macklin	C_10_H_14_	1273	2900
Naphthalene	91-20-3	≥ 99.5%	Macklin	C_10_H_8_	1635	2070
**Volatile phenol**						
4-Methylguaiacol	93-51-6	>99.0%	Sigma-Aldrich	C_8_H_10_O_2_	1860	2820
*o*-Cresol	95-48-7	>99.0%	Sigma-Aldrich	C_7_H_7_O	1913	4980
4-Propylguaiacol	2785-87-7	>99.0%	Sigma-Aldrich	C_10_H_14_O_2_	2011	5370
4-Vinylphenol	2628-17-3	>95.0%	Sigma-Aldrich	C_8_H_8_O	2306	2540
**Sulfide**						
3-(Methylthio)propanol	505-10-2	≥99.0%	Macklin	C_4_H_10_OS	1618	6600
**Internal standard**						
4-Methyl-2-pentanol	108-11-2	≥98.0%	CNW^f^	C_6_H_14_O	1065	1000

^a^Shanghai Macklin Biochemical Co., Ltd (Shanghai, China).

^b^Aladdin Bio-Chem Technology (Shanghai, China).

^c^Adamas Reagent, Co., Ltd. (Shanghai, China).

^d^TCI Development Co., Ltd. (Shanghai, China).

^e^Sigma-Aldrich (St. Louis, MO, USA).

^f^CNW Technologies GmbH (Duesseldorf, Germany).

^g^Bide Pharmatech Ltd. (Shanghai, China).

^h^The contents of spiked standard mixtures used in direct liquid introduction method.

##### Wine Samples collection

Three kinds of commercial fruit wines (blueberry wine, B, goji berry wine, G and hawthorn wine, H) purchased from retail stores in China were used for the establishment of HRMS library. All blueberry samples were with an alcohol content of 12% v/v (percent by volume). Three blueberry wines were received from Beiyushidai, including blueberry dry wine produced in 2019 (B1) and 2017 (B2) and blueberry semi-dry wine produced in 2019 (B3). A blueberry dry wine (B4) was produced by Shenghua in 2019. Another blueberry dry wine (B5) produced in 2019 was provided by Yicunshanye. Goji berry semi-dry wine (G1) was produced by Ningxiahong in 2019, with an alcohol content of 7% v/v. Four batches of goji berry dry wine (G2-G5) produced by Senmiao in 2017 were with an alcohol content of 11% v/v. G6 was made by our laboratory in 2016 with an alcohol content of 11% v/v. All hawthorn wine samples were semi-dry wines from Shengbali. H1 and H2 produced in 2019 were with an alcohol content of 12% v/v. The other H3-H5 were produced in 2020 with an alcohol content of 13% v/v from Shengbali.

##### Preparation of the spiked mixture

The direct liquid introduction method was used to determine the mass spectral information of the target compound. The standard mixtures (Mixture 1 with 24 esters, Mixture 2 with 6 carbonyl compounds and 8 lactones and 6 acids, Mixture 3 with10 high alcohols and 6 furans and 5 pyrazines, Mixture 4 with 5 terpenes and 4 benzenes and 4 volatile phenols and 1 sulfide) were prepared to extract. The mother solution of each compound was dissolved in ethanol at higher concentration. Each standard mixtures were mixed by the mother solution of compounds according to the concentrations (Table 1).The standard mixtures were diluted with dichloromethane to volume in a 10-mL volumetric flask. 1 μL of each mixture was injected. The split mode was applied with a split ratio of 10:1. The liquid injection was performed using the TriPlus RSH autosampler (Thermo Fisher Scientific, Bremen, Germany).

##### Extraction of volatile compounds in wine samples

Headspace solid-phase microextraction (HS-SPME) was used to extract the volatile compounds from fruit wines. 5 mL of wine samples mixed with 1.00 g NaCl and 10 μL of internal standard (1.077 g/L 4-methyl-2-pentanol) were prepared in a 20 mL glass vial. The sample vials were stirred and heated at 60 °C for 30 min. Then the preconditioned fiber (50/30 μm Divinylbenzene/Carboxen/Polydimethylsiloxane (DVB/CAR/PDMS)) was used to absorb the volatile compounds in the headspace of the sample via for 30 min at 60 °C. After absorption, the fiber was inserted into the GC injection port for desorbing at 250 °C for 10 min. Two technical replicates were performed for each sample. Automatic headspace solid-phase microextraction was performed on the TriPlus RSH autosampler.

##### GC-Orbitrap-MS analysis

A Thermo Scientific Trace 1300 gas chromatography equipped with a Thermo Scientific Q-Exactive Orbitrap mass spectrometer (GC-Orbitrap MS, Thermo Scientific, Bremen, Germany) was used for detection. The spiked mixture was performed under the following GC-Orbitrap-MS conditions. A TG-WAXMS 30 m × 0.25 mm × 0.25 μm (Thermo Scientific, Bremen, Germany) was used to separate analytes. Helium was used as the carrier gas (1.2 mL/min). The oven temperature program was set as follows: 40 °C held for 5 min, then heated to 180 °C at 3 °C/min, finally increased from 180 °C to 240 °C at 30 °C/min and hold 15 min. The wine samples were performed under the following GC-Orbitrap-MS conditions. A DB-WAX 30 m × 0.25 mm × 0.25 μm (J&W Scientific, Folsom, CA, USA) was used to separate the volatile compounds under a 1.2 mL/min flow rate of helium (carrier gas).The oven temperature program was set as follows: 40°C held for 5 min, then heated to 180°C at 3 °C /min, finally increased from 180 °C to 250 °C at 30 °C/min and hold 10 min.

The Orbitrap-MS operated in full-scan MS acquisition mode (m/z 33–350). The ion source was maintained at 280 °C with an MSD transfer line temperature of 230 °C. Positive ion-electron ionization (EI) was used at 70 electron volts (eV) in Orbitrap-MS.

##### Identification of the compounds

**Fig. 1:**
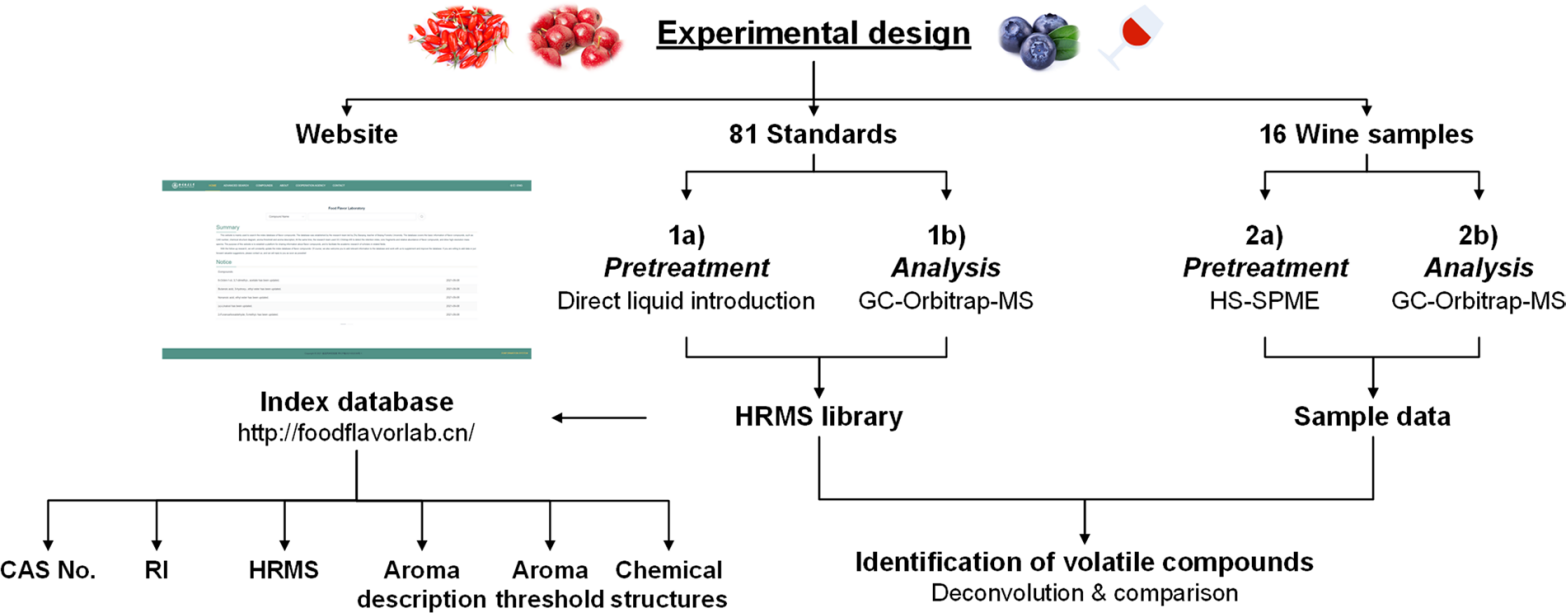
Flowchart of the experimental design.

Retention indices (RI) were calculated from the retention times of C6-C24 n-alkanes under the same chromatographic and mass spectrometric conditions. The high-solution mass spectrums of volatile compounds were collected in different standard mixtures. Then, the qualitative determination of target compounds in fruit wines was performed by the match of the retention time and ion fragments in samples and standards.The experimental design and analysis pipeline are shown in Fig. 1.

## Data Records

A total of 36 original data files were stored in MetaboLights^47^, including 4 standard mixtures and 32 wine samples (two technical replicates).

## Technical Validation

Two technical replicates were performed on each wine sample. The qualitative determination of target volatile compounds in fruit wines was shown in Table 3.

## Usage Notes

**Fig. 2:**
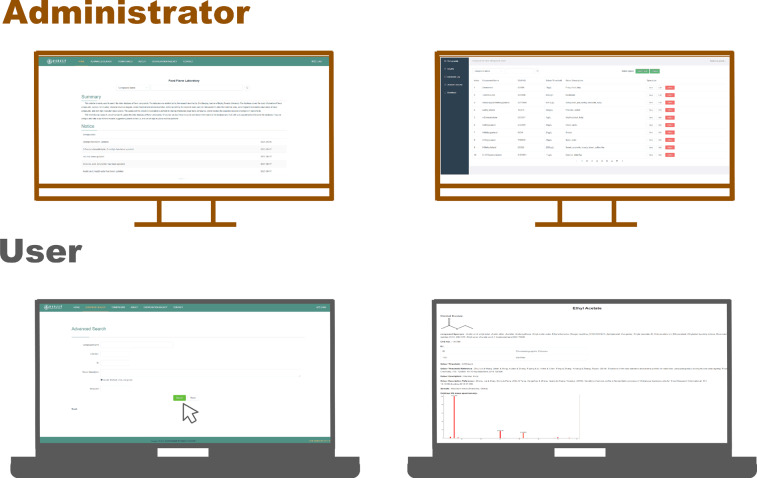
The web page of the database website (http://foodflavorlab.cn/) including the home page, upload page, search page and result page.

**Fig. 3:**
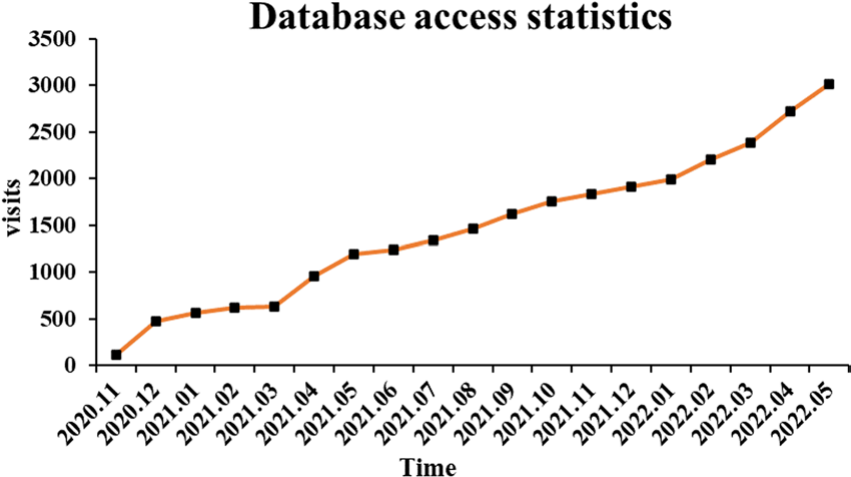
The page view (PV) of database website (http://foodflavorlab.cn/).

The HRMS library of volatile compounds was shown on the database website (http://foodflavorlab.cn/), including HRMS spectrum, exact ion fragment, relative abundance, RI, CAS number, chemical structure diagram, aroma description and aroma threshold (ortho-nasally). Table 1 showed CAS No., formula and RI of each target volatile compound. The information of standards and contents of spiked mixtures were shown in Table 1. Table 2 showed elemental composition judgments, exact ion fragments and error mass of each target volatile compound. Table 3 showed the qualitative determination of target volatile compounds in blueberry wine, goji berry wine and hawthorn wine. Figure 2 showed the web page of the database website (http://foodflavorlab.cn/) including the home page, upload page, search page and result page. Figure 3 showed the page view (PV) of the database website (http://foodflavorlab.cn/) from Nov. 2020 to May. 2022.

**Table 2:** The qualitative and quantitative information of target volatile compounds.

Compounds	Precursor ions			Quantifier ions			Qualifier ions		
	Exact mass (m/z)	Molecular formula	Error mass (ppm^a^)	Exact mass (m/z)	Molecular formula	Error mass (ppm)	Exact mass (m/z)	Molecular formula	Error mass (ppm)
**Ester**									
Ethyl butanoate				43.05422	C_3_H_7_	−0.99526	88.05202	C_4_H_8_O_2_	0.7668
Ethyl 2-methylbutanoate				74.03639	C_3_H_6_O_2_	0.2198	102.0677	C_5_H_10_O_2_	0.41588
Ethyl isovalerate				57.06997	C_4_H_9_	0.34743	61.0285	C_2_H_5_O_2_	0.15943
Isoamyl acetate				43.01782	C_2_H_3_O	−1.06298	55.05433	C_4_H_9_	0.6148
Methyl caproate				43.01782	C_2_H_3_O	−0.70828	74.03639	C_3_H_6_O_2_	0.52895
Ethyl hexanoate				43.05422	C_3_H_7_	−0.99526	73.02851	C_3_H_5_O_2_	0.70783
Ethyl heptanoate				73.02854	C_3_H_5_O_2_	0.49889	88.05192	C_4_H_8_O_2_	0.42009
Ethyl lactate				45.03354	C_2_H_5_O	1.30819	56.0621	C_4_H_8_	0.9174
Heptyl acetate				43.01778	C_2_H_3_O	−0.17621	70.07773	C_5_H_10_	0.8118
Methyl octanoate				43.01782	C_2_H_3_O	−0.70828	74.03639	C_3_H_6_O_2_	0.73505
Ethyl caprylate				73.02845	C_3_H_5_O_2_	−0.44136	101.05977	C_5_H_9_O_2_	−0.43741
Ethyl 3-hydroxybutyrate				43.01778	C_2_H_3_O	−0.6196	71.01285	C_3_H_3_O_2_	1.29569
Ethyl nonanoate				73.02845	C_3_H_5_O_2_	−0.54583	101.05977	C_5_H_9_O_2_	−0.51291
Ethyl 2-hydroxy-4-methylpentanoate				69.06999	C_5_H_9_	0.12138	45.03355	C_2_H_5_O	1.22348
Ethyl caprate				73.02853	C_3_H_5_O_2_	0.39441	61.0285	C_2_H_5_O_2_	0.28445
Ethyl succinate				101.02348	C_4_H_5_O_3_	0.02484	73.02853	C_3_H_5_O_2_	0.60336
Methyl salicylate	152.04683	C_8_H_8_O_3_	0.22088	120.02077	C_7_H_4_O_2_	0.28082	92.02578	C_6_H_4_O	0.15454
Ethyl benzeneacetate	164.08322	C_10_H_12_O_2_	0.24546	91.05439	C_7_H_7_	−0.13544	136.05219	C_8_H_8_O_2_	0.66442
Ethyl salicylate	166.06245	C_9_H_10_O_3_	0.05133	120.02077	C_7_H_4_O_2_	0.40795	92.02578	C_6_H_4_O	0.15454
Ethyl hydrocinnamate	178.09898	C_11_H_14_O_2_	0.85652	104.06216	C_8_H_8_	0.34761	105.06997	C_8_H_9_	0.15241
Ethyl cinnamate	176.08331	C_11_H_12_O_2_	0.96579	131.04938	C_9_H_7_O	0.98604	103.05436	C_8_H_7_	0.62066
Monoethyl succinate				101.02348	C_4_H_5_O_3_	0.10036	73.02853	C_3_H_5_O_2_	0.60336
**Carbonyl compounds**									
(*E*)-2-Hexenal				83.04919	C_5_H_7_O	0.17795	69.03339	C_6_H_9_O	0.46658
(*E*)-2-Heptenal				83.04919	C_5_H_7_O	0.54542	41.03839	C_3_H_5_	−3.22212
(*E*)-2-Octenal				83.04919	C_5_H_7_O	0.17795	41.03839	C_3_H_5_	−4.80235
(*E*,*E*)-2,4-Heptadienal				81.03347	C_5_H_5_O	−0.26157	109.0647	C_7_H_9_O	0.11559
(*E*,*Z*)-2,6-Nonadienal				41.03839	C_3_H_5_	−4.33758	70.04136	C_4_H_6_O	−0.48152
Benzeneacetaldehyde	120.05711	C_8_H_8_O	1.14129	91.05439	C_7_H_7_	0.61866	92.06208	C_7_H_8_	0.31004
**High alcohols**									
Isobutanol				41.0384	C_3_H_5_	−4.52349	45.0336	C_2_H_5_O	2.32468
Isoamylol				57.0699	C_4_H_9_	0.41428	70.07784	C_5_H_10_	0.37632
1-Pentanol				57.06991	C_4_H_9_	0.54796	70.07784	C_5_H_10_	0.59406
2-Heptanol				45.03354	C_2_H_5_O	0.88465	83.08566	C_6_H_11_	0.5339
3-Octenol				57.03355	C_3_H_5_O	0.49786	85.06478	C_5_H_9_O	0.50696
1-Heptanol				43.05422	C_2_H_3_O	−1.06298	70.07338	C_5_H_10_	0.48519
2-Nonanol				105.03364	C_7_H_5_O	0.74249	122.03642	C_7_H_6_O_2_	0.75852
1-Octanol				69.06999	C_5_H_9_	0.23184	55.05433	C_4_H_7_	0.3996
2-Phenylethanol	122.07275	C_8_H_10_O	1.06311	91.05439	C_7_H_7_	0.95382	92.06208	C_7_H_8_	−0.51868
2-Phenoxyethanol				108.05687	C_7_H_8_O	−0.89706	94.04132	C_6_H_6_O	0.04701
**Lactone**									
γ-Undecalactone				85.02853	C_4_H_5_O_2_	0.69766	95.0493	C_6_H_7_O	0.95817
δ-Octalactone				99.04407	C_5_H_7_O_2_	−0.11627	71.04915	C_4_H_7_O	0.74492
γ-Octalactone				85.02851	C_4_H_5_O_2_	−0.10989	57.03359	C_3_H_5_O	0.49786
Pantolactone				71.04915	C_4_H_7_O	0.10063	43.05414	C_3_H_7_	−2.23569
γ-Decalactone				85.02853	C_4_H_5_O_2_	0.1593	95.0493	C_6_H_7_O	0.47656
Sotolon	128.04693	C_6_H_8_O_3_	0.18604	83.04919	C_5_H_7_O	0.08357	55.05427	C_4_H_7_	0.81287
γ-Nonalactone				85.02851	C_4_H_5_O_2_	−0.02016	57.03359	C_3_H_5_O	0.36409
**Acid**									
Butanoic acid				60.02063	C_2_H_4_O_2_	0.56154	73.02845	C_3_H_5_O_2_	0.39441
Hexanoic acid				73.02853	C_3_H_5_O_2_	0.18547	60.02069	C_2_H_4_O_2_	0.39361
Ethylhexanoic acid				73.02853	C_3_H_5_O_2_	0.18547	87.04422	C_4_H_7_O_2_	0.48125
Octanoic acid				73.02853	C_3_H_5_O_2_	0.18547	101.05988	C_5_H_9_O_2_	0.6195
Decanoic acid				73.02844	C_3_H_5_O_2_	0.49889	101.05976	C_5_H_9_O_2_	0.54401
Benzoic acid	122.03632	C_7_H_6_O_2_	0.75852	105.03364	C_7_H_5_O	0.66985	122.03642	C_7_H_6_O_2_	0.75852
**Pyrazine**									
3-Isopropyl-2-methoxypyrazine	152.09455	C_8_H_12_ON_2_	0.50811	137.071	C_7_H_9_ON_2_	0.50114	124.06324	C_6_H_8_ON_2_	0.86187
2-sec-Butyl-3-Methoxypyrazine	166.10973	C_9_H_14_ON_2_	−1.99624	138.07886	C_7_H_10_ON_2_	0.56568	124.06321	C_6_H_8_ON_2_	0.75882
5-Ethyl-2,3-dimethylpyrazine	136.0996	C_8_H_12_N_2_	0.64728	135.0918	C_8_H_11_N_2_	0.714	121.07612	C_7_H_9_N_2_	−0.02603
2-Isobutyl-3-methoxypyrazine	166.11008	C_9_H_14_ON_2_	0.08705	124.0632	C_6_H_8_ON_2_	0.58057	95.06044	C_5_H_7_N_2_	−0.08289
Acetylpyrazine	122.04759	C_6_H_6_ON_2_	0.53454	94.0526	C_5_H_6_N_2_	0.35341	80.03695	C_4_H_4_N_2_	0.43185
**Furan**									
Furfural	96.02053	C_5_H_4_O_2_	−0.84083	95.01279	C_5_H_3_O_2_	0.16541	39.02277	C_3_H_3_	−3.43066
Acetylfuran	110.03637	C_6_H_6_O_2_	0.42523	95.01281	C_5_H_3_O_2_	0.64721	43.01782	C_2_H_3_O	−0.41717
5-Methylfurfural	110.03625	C_6_H_6_O_2_	−0.47613	109.02855	C_6_H_5_O_2_	0.68404	53.03864	C_4_H_5_	1.24689
Ethyl 2-furoate	140.04697	C_7_H_8_O_3_	0.56667	95.01279	C_5_H_3_O_2_	−0.07548	112.01554	C_5_H_4_O_3_	−0.07007
Furfuryl alcohol	98.03629	C_5_H_6_O_2_	0.01035	97.02851	C_5_H_5_O_2_	0.29686	81.0336	C_5_H_5_O	0.11503
5-Hydroxymethylfurfural	126.03131	C_6_H_6_O_3_	0.34424	97.02849	C_5_H_5_O_2_	0.29686	69.03357	C_4_H_5_O	0.5771
**Terpenes**									
D-Limonene	136.1252	C_10_H_16_	1.65914	93.07005	C_7_H_9_	1.89353	121.10146	C_9_H_13_	1.60836
Terpinolene	136.12471	C_10_H_16_	0.4261	121.10132	C_9_H_13_	0.22236	93.06999	C_7_H_9_	0.25403
β-Linalool				93.07005	C_7_H_9_	0.41798	69.03339	C_5_H_9_	0.3423
Citronellyl acetate				81.06996	C_6_H_9_	0.00931	95.08559	C_7_H_11_	−0.17538
β-Ionone				177.12753	C_12_H_17_O	0.28057	178.13091	C_12_H_17_O	−2.13518
**Benzene**									
*o*-Xylene	106.07779	C_8_H_10_	−0.11101	91.05439	C_7_H_7_	0.03214	103.05429	C_8_H_7_	0.62066
Styrene	104.0621	C_8_H_8_	−0.09229	104.0621	C_8_H_8_	−0.09229	78.04652	C_6_H_6_	0.78457
*p*-Cymene	134.10954	C_10_H_14_	0.39181	119.0857	C_9_H_11_	0.05216	115.0543	C_9_H_7_	0.68855
Naphthalene	128.06218	C_10_H_8_	0.04416	128.06218	C_10_H_8_	0.04416	129.06557	C_10_H_8_	−3.16989
**Volatile phenol**									
4-Methylguaiacol	138.06754	C_8_H_10_O_2_	0.04814	138.06754	C_8_H_10_O_2_	0.04814	123.04407	C_7_H_7_O_2_	−0.00386
*o*-Cresol	107.04918	C_7_H_7_O	0.20933	107.04918	C_7_H_7_O	0.20933	79.05427	C_6_H_7_	0.42305
4-Propylguaiacol	166.09877	C_10_H_14_O_2_	−0.18399	137.05968	C_8_H_9_O_2_	0.01147	122.03631	C_7_H_6_O_2_	0.19587
4-Vinylphenol	120.057	C_8_H_8_O	0.14583	120.057	C_8_H_8_O	0.14583	91.05425	C_7_H_7_	0.19972
**Sulfide**									
3-(Methylthio)propanol	106.04483	C_7_H_6_O	3.52157	106.04483	C_7_H_6_O	3.52157	88.03425	C_7_H_4_	3.50426
**Internal standard**									
4-Methyl-2-pentanol				45.03355	C_2_H_5_O	0.79994			

^a^ppm means parts per million mass error.

**Table 3:** The qualitative determination of target volatile compounds in goji berry wines, blueberry wines and hawthorn wines.

Compounds	B1	B2	B3	B4	B5	G1	G2	G3	G4	G5	G6	H1	H2	H3	H4	H5
**Ester**																
Ethyl butanoate	√	√	√	√	√	√	√	√	√	√	√	√	√	√	√	√
Ethyl 2-methylbutanoate	√	√	√	√	√	√	√	√	√	√	√	√	√	√	√	√
Ethyl isovalerate	√	√	√	√	√	√	√	√	√	√	√	√	√	√	√	√
Isoamyl acetate	√	√	√	√	√	√	√	√	√	√	√	√	√	√	√	√
Methyl caproate	√	√	√	√	√	√	√	√	√	√	√	√	√	√	√	√
Ethyl hexanoate	√	√	√	√	√	√	√	√	√	√	√	√	√	√	√	√
Ethyl heptanoate	√	√	√	√	√	√	√	√	√	√	√	√	√	√	√	√
Ethyl lactate	√	√	√	√	√	√	√	√	√	√	√	√	√	√	√	√
Heptyl acetate	√	√	√	√	√	√	√	√	√	√	nd	√	nd	nd	nd	nd
Methyl octanoate	√	√	√	√	√	√	√	√	√	√	√	√	√	√	√	√
Ethyl caprylate	√	√	√	√	√	√	√	√	√	√	√	√	√	√	√	√
Ethyl 3-hydroxybutyrate	√	√	√	√	√	√	√	√	√	√	√	√	√	√	√	√
Ethyl nonanoate	√	√	√	√	√	√	√	√	√	√	√	√	√	√	√	√
Ethyl 2-hydroxy-4-methylpentanoate	√	√	√	√	√	√	√	√	√	√	√	√	√	√	√	√
Ethyl caprate	√	√	√	√	√	√	√	√	√	√	√	√	√	√	√	√
Ethyl succinate	√	√	√	√	√	√	√	√	√	√	√	√	√	√	√	√
Methyl salicylate	√	√	√	√	√	√	√	√	√	√	√	√	√	√	nd	√
Ethyl benzeneacetate	√	√	√	√	√	√	√	√	√	√	√	√	√	√	√	√
Ethyl salicylate	√	√	√	√	√	√	√	√	√	√	√	√	√	√	√	√
Ethyl hydrocinnamate	√	√	√	√	√	√	√	√	√	√	√	√	√	√	√	√
Ethyl cinnamate	√	√	√	√	√	√	√	√	√	√	√	√	√	√	√	√
Monoethyl succinate	√	√	√	√	√	√	√	√	√	√	√	√	√	√	√	√
**Carbonyl compounds**																
(*E*)-2-Hexenal	√	√	√	√	√	√	√	√	√	√	√	√	√	√	√	√
(*E*)-2-Heptenal	√	√	√	√	√	√	√	√	√	√	√	√	√	√	√	√
(*E*)-2-Octenal	√	√	√	√	√	√	√	√	√	√	√	√	√	√	√	√
(*E*,*E*)-2,4-Heptadienal	√	√	√	√	√	√	√	√	√	√	√	√	√	√	√	√
(*E*,*Z*)-2,6-Nonadienal	√	√	√	√	√	√	√	√	√	√	√	√	√	√	√	√
Benzeneacetaldehyde	√	√	√	√	√	√	√	√	√	√	√	√	√	√	√	√
**High Alcohols**																
Isobutanol	√	√	√	√	√	√	√	√	√	√	√	√	√	√	√	√
Isoamylol	√	√	√	√	√	√	√	√	√	√	√	√	√	√	√	√
1-Pentanol	√	√	√	√	√	√	√	√	√	√	√	√	√	√	√	√
2-Heptanol	√	√	√	√	√	√	√	√	√	√	√	√	√	√	√	√
3-Octenol	√	√	√	√	√	√	√	√	√	√	√	√	√	√	√	√
1-Heptanol	√	√	√	√	√	√	√	√	√	√	√	√	√	√	√	√
2-Nonanol	√	√	√	√	√	√	√	√	√	√	√	√	√	√	√	√
1-Octanol	√	√	√	√	√	√	√	√	√	√	√	√	√	√	√	√
2-Phenylethanol	√	√	√	√	√	√	√	√	√	√	√	√	√	√	√	√
2-Phenoxyethanol	√	√	√	√	√	√	√	√	√	√	√	√	√	√	√	√
**Lactone**																
γ-Undecalactone	√	√	√	√	√	√	√	√	√	√	√	√	√	√	√	√
δ-Octalactone	√	√	√	√	√	√	√	√	√	√	√	√	√	√	√	√
γ-Octalactone	√	√	√	√	√	√	√	√	√	√	√	√	√	√	√	√
Pantolactone	√	√	√	√	√	√	√	√	√	√	√	√	√	√	√	√
γ-Decalactone	√	√	√	√	√	√	√	√	√	√	√	√	√	√	√	√
Sotolon	√	√	√	√	nd	nd	√	nd	nd	√	nd	nd	nd	nd	nd	nd
γ-Nonalactone	√	√	√	√	√	√	√	√	√	√	√	√	√	√	√	√
**Acid**																
Butanoic acid	√	√	√	√	√	√	√	√	√	√	√	√	√	√	√	√
Hexanoic acid	nd	√	nd	√	√	√	√	√	√	√	√	√	√	√	√	√
Ethylhexanoic acid	nd	√	nd	√	√	√	√	√	√	√	√	√	√	√	√	√
Octanoic acid	√	√	√	√	√	√	√	√	√	√	√	√	√	√	√	√
Decanoic acid	√	√	√	√	√	√	√	√	√	√	√	√	√	√	√	√
Benzoic acid	√	√	√	√	√	√	√	√	√	√	√	√	√	√	√	√
**Pyrazine**																
3-Isopropyl-2-methoxypyrazine	√	√	√	√	√	√	√	√	√	nd	√	√	√	√	nd	√
2-sec-Butyl-3-Methoxypyrazine	√	√	√	√	nd	√	√	nd	√	nd	nd	√	√	nd	√	√
5-Ethyl-2,3-dimethylpyrazine	√	√	√	√	√	√	√	√	√	√	√	√	√	√	√	√
2-Isobutyl-3-methoxypyrazine	√	√	√	√	√	√	√	√	√	√	√	√	√	√	√	√
Acetylpyrazine	nd	nd	√	√	nd	nd	√	√	√	nd	nd	√	√	√	√	√
**Furan**																
Furfural	√	√	√	√	√	√	√	√	√	√	√	√	√	√	√	√
Acetylfuran	√	√	√	√	√	√	√	√	√	√	√	√	√	√	√	√
5-Methylfurfural	√	√	√	√	√	√	√	√	√	√	√	√	√	√	√	√
Ethyl 2-furoate	√	√	√	√	√	√	√	√	√	√	√	√	√	√	√	√
Furfuryl alcohol	√	√	√	√	√	√	√	√	√	√	√	√	√	√	√	√
5-Hydroxymethylfurfural	√	√	√	√	√	√	√	√	√	√	√	√	√	√	√	√
**Terpenes**																
D-Limonene	√	√	√	√	√	√	√	√	√	√	√	√	√	√	√	√
Terpinolene	√	√	√	√	√	√	√	√	√	√	√	√	√	√	√	nd
β-Linalool	√	√	√	√	nd	nd	√	√	√	√	nd	nd	nd	nd	√	nd
Citronellyl acetate	√	√	√	√	√	√	√	√	√	√	√	√	√	√	√	√
β-Ionone	√	√	√	nd	√	√	√	√	√	√	√	√	nd	√	nd	√
**Benzene**																
*o*-Xylene	nd	nd	nd	nd	nd	nd	nd	nd	√	nd	nd	nd	nd	√	√	√
Styrene	√	√	√	√	√	√	√	√	√	√	√	√	nd	√	√	√
*p*-Cymene	√	√	√	√	√	√	√	√	√	√	√	√	√	√	√	√
Naphthalene	√	√	√	√	√	√	√	√	√	√	√	√	√	√	√	√
**Volatile phenol**																
4-Methylguaiacol	√	√	√	√	√	√	√	√	√	√	√	√	√	√	√	√
*o*-Cresol	√	√	√	√	√	√	√	√	√	√	√	√	√	√	√	√
4-Propylguaiacol	√	√	√	√	√	√	√	√	√	√	√	√	√	√	√	√
4-Vinylphenol	√	√	√	√	√	nd	√	√	√	√	√	nd	nd	nd	nd	nd
**Sulfide**																
3-(Methylthio)propanol	√	√	√	nd	√	√	√	√	√	√	√	√	√	√	√	√

‘B’ represent blueberry wine, ‘G’ represent goji berry wine, ‘H’ represent hawthorn wine.

## Data Availability

The Processing setup, Quan browser and Qual browser (Thermo Fisher Scientific, Les Ulis, France) in Xcalibur version 4.1 and Thermo Scientific TraceFinder (version 4.1) were used for collecting the HRMS library of volatile compounds. The structures of the volatile compounds were drawn using ChemDraw Professional 17.0 (Cambridgesoft, USA). High-resolution mass spectrums are plotted using Python (version 3.7).
